# Silvopastoral systems and remnant forests enhance carbon storage in livestock-dominated landscapes in Mexico

**DOI:** 10.1038/s41598-022-21089-4

**Published:** 2022-10-06

**Authors:** Deb Raj Aryal, Danilo Enrique Morales-Ruiz, Susana López-Cruz, César Noe Tondopó-Marroquín, Alejandra Lara-Nucamendi, José Antonio Jiménez-Trujillo, Edwin Pérez-Sánchez, Juan Edduardo Betanzos-Simon, Francisco Casasola-Coto, Alejandra Martínez-Salinas, Claudia Janeth Sepúlveda-López, Roselia Ramírez-Díaz, Manuel Alejandro La O Arias, Francisco Guevara-Hernández, René Pinto-Ruiz, Muhammad Ibrahim

**Affiliations:** 1 Consejo Nacional de Ciencia y Tecnología, 03940 Ciudad de México, Mexico; 2 Universidad Autónoma de Chiapas, Facultad de Ciencias Agronómicas, Villaflores, 30470 Chiapas, Mexico; 3 CATIE-Centro Agronómico Tropical de Investigación y Enseñanza, 30501 Turrialba, Costa Rica

**Keywords:** Carbon cycle, Climate-change mitigation, Ecosystem services, Biodiversity

## Abstract

A large area of the terrestrial land surface is used for livestock grazing. Trees on grazing lands provide and can enhance multiple ecosystem services such as provisioning, cultural and regulating, that include carbon sequestration. In this study, we assessed the above- and belowground carbon stocks across six different land-uses in livestock-dominated landscapes of Mexico. We measured tree biomass and soil organic carbon (SOC) stocks in fodder banks, live fences, pasturelands with dispersed trees, secondary forests, and primary forests from three different geographical regions and compared them with conventional open pasturelands respectively. We also calculated tree diversity indices for each land-use and their similarity with native primary forests. The aboveground woody biomass stocks differed significantly between land-uses and followed the gradient from less diverse conventional open pasturelands to silvopastoral systems and ecologically complex primary forests. The SOC stocks showed a differential response to the land-use gradient dependent on the study region. Multivariate analyses showed that woody biomass, fine root biomass, and SOC concentrations were positively related, while land-use history and soil bulk density showed an inverse relationship to these variables. Silvopastoral systems and forest remnants stored 27–163% more carbon compared to open pasturelands. Our results demonstrate the importance of promoting appropriate silvopastoral systems and conserving forest remnants within livestock-dominated landscapes as a land-based carbon mitigation strategy. Furthermore, our findings also have important implications to help better manage livestock-dominated landscapes and minimize pressures on natural protected areas and biodiversity in the hotspots of deforestation for grassland expansion.

## Introduction

Land-use change significantly alters the global biogeochemical cycles and contributes to climate change^1^. Between 1850 and 2015, global land-use change emitted an estimated 145 ± 16 Pg of carbon (C) to the atmosphere, counting the loss of biomass and soil organic carbon (SOC)^2^. Land conversion from forest to agriculture (i.e., crops and pasturelands) was one of the main drivers causing such C emissions^3–5^. Ongoing land-use change from forest to crop and grasslands not only emits C due to the loss of vegetation but also depletes up to 60% of SOC^6,7^. Estimate from historical land-use change accounts for a loss of 133–135 Pg SOC^8,9^. Between 2009 and 2018, land-use change contributed on average 14% (1.5 ± 0.7 Pg C year^−1^) of the total C emissions globally^10^. However, terrestrial ecosystems annually remove 3.2 ± 0.6 Pg of C from the atmosphere and store it in biomass and soils offsetting 29% of the anthropogenic C emissions^10,11^. Owing to the contribution, the implementation of agroforestry practices (e.g., silvopastoral systems) can enhance biomass as well as soil C storage in crop and pasturelands^12–16^.

Currently, about 37% of ice-free land surface worldwide is used for livestock grazing, which is three times larger than cropland area^17,18^. In Latin America alone, the area dedicated to pastureland increased nearly 2.5 times between 1850 and 2015, expanding from 229 to 564 million ha, while forest area decreased 25%, from 1248 to 932 million ha^2^. Additionally, it is estimated that more than one-third of the pastureland in Latin America is in a state of degradation and exhibits low resilience to climate variability^19–21^. In Mexico, 80.3 million ha of land are currently used as permanent and cultivated pasture lands^17^. Furthermore, during the years between 1985 and 2011, induced pastureland establishment accounted for 41% of the total native vegetation (mainly scrubland and tropical forest) loss, with the expansion of bovine livestock grazing currently considered one of the main drivers of deforestation in South and Southeastern Mexico^22–25^. Finally, conversion of native vegetation to pasturelands has caused the loss of 30 to 50% of the SOC stocks (0–60 cm depth) nationwide^26^.

Lands devoted to livestock production can be converted to treed ecosystems like silvopasture to augment C storage at regional and global scales^27–30^. Global soils have the potential to sequester an additional 1.62 Pg C year^−1^, of which 47% corresponds to SOC enhancement in agriculture and grasslands through a wide range of soil management practices^14^. Besides the increase in above- and belowground C storage, silvopastoral systems (SPS) create habitat for wildlife and promote biodiversity conservation through landscape connectivity^31–33^. Compared to conventional grass monoculture, SPS also ensures greater ecosystem resilience and decreases climate vulnerability^34–36^.

Extensive grass monoculture practices for animal production have high environmental footprints^37–40^. Inappropriate management and overgrazing lead to land degradation^41–43^. Globally, 93% of pasturelands have an aboveground C density of less than 5 Mg ha^−1^^28^. Hence, C enhancement through the implementation of SPS, and conservation of forest remnants within farmlands are the most promising nature-based solutions to mitigate greenhouse gas (GHG) emissions and increase ecosystem services from the livestock farmlands^27,44,45^. Converting conventional open pasturelands to SPS increases the accrual of atmospheric CO_2_ while controlling deforestation for the expansion of pure grasslands reduces CO_2_ emissions through C retention within the landscape^46,47^. Furthermore, SPS also contributes to reducing or buffering CO_2_ emissions from soil respiration compared to open pasturelands^48,49^. Additional benefits of C enhancement through SPS include: nutrient cycling and soil fertility improvement, increasing biological diversity, reduce soil erosion, increase forage production, and regulating heat stress to grazing animals through tree shades^50–52^.

Silvopasture is an animal-agroforestry where woody perennials, grasses, and animals interact biologically and economically in the same land unit^53,54^. There are multiple combinations of woody perennials and grasses in space and time to optimize resources and diversify production as well as ecological benefits^55–57^. Dispersed trees in pastures, woody perennials with grasses in alleys, live fences (shelterbelts), fodder banks, and grasses under tree plantations are some examples of SPS^53,58^. In many parts of the world, forest browsing is also a common animal agroforestry practice. SPS has been promoted throughout Latin America and in other parts of the world due to its multiple benefits, including productivity increase, animal wellbeing, C sequestration, and biodiversity conservation^59–62^.

Earlier studies have shown that C storage in SPS is highly variable and depends greatly on local ecological conditions, land-use history, and management intensity^41,63–65^. Some have demonstrated the value of using land-use intensity gradients to show the impacts of forested ecosystems simplification on biodiversity^56,66–68^, fewer studies have used this approach to explore the variations in C storage in livestock-dominated landscapes, particularly comparing simplified open pasturelands with complex silvopasture and forested systems.

**Figure 1:**
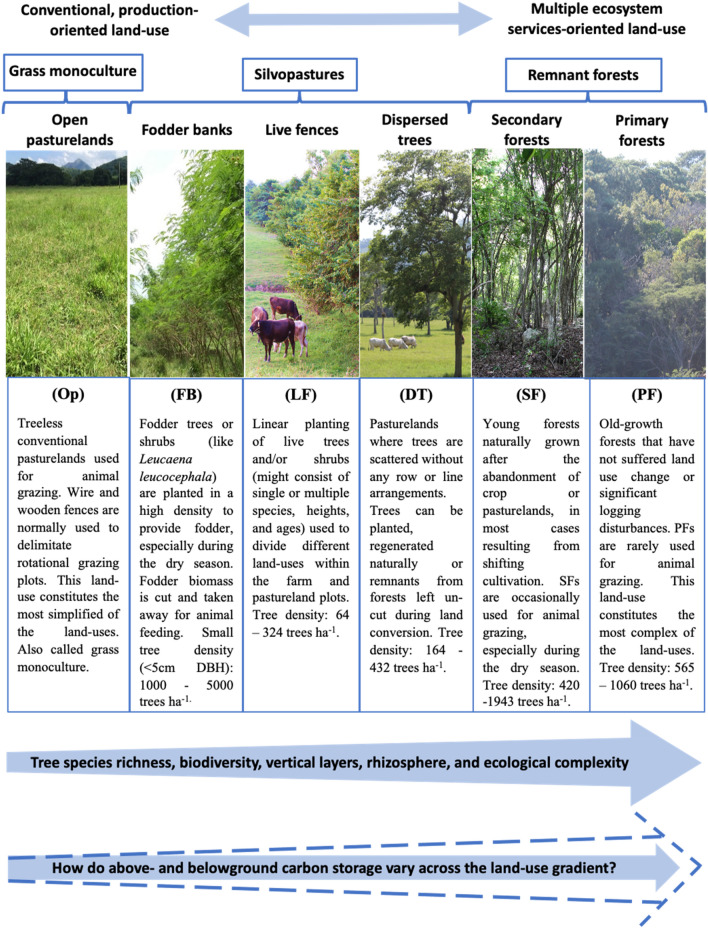
Illustration and description of the land-use gradient from conventional open pasturelands (OP), fodder banks, (FB) live fences (LF), pasturelands with dispersed trees (DT), secondary forests (SF) to primary forests (PF) selected for carbon monitoring. Land-uses followed an intensity gradient from ecologically simple to complex systems. The photographs were taken by Deb Raj Aryal from Chiapas (OP, FB, DT, PF) and Campeche (SF), Mexico. The animals in the images were to represent that the lands we sampled are used for animal grazing.

In this context, our understanding of the variations in C storage among different land-use systems in a gradient from open pasture to silvopasture and forest remnants is limited. Carbon estimations by ecological zone default values do not cover the finer scale differences between land-use within the same landscape^28^. Finer scale evaluations of C densities within the landscape are required to better understand the regional ecosystem change processes and articulate them to the land management decisions. Effective formulation and implementation of strategies to enhance C storage in lands used for livestock production require detailed knowledge regarding the quantities of C that can be stored across different land-uses including SPS and their relationship with land-use history and soil properties. In Mexico, studies related to C sequestration in livestock-dominated landscapes are mostly limited to only one geographical region or covering only one or a few land-use types^69–71^. Accounting C storage in a gradient of land-uses from structurally simple open pastures to ecologically more complex systems like multi-species silvopasture and native forests (Fig. 1) are important to better understand the ecological processes and GHG mitigation potential. Here, we evaluated the tree species richness, diversity, and C stocks across a land-use gradient, from conventional pasture monoculture and silvopasture to primary forest in livestock-dominated landscapes. We quantified C stocks in above and belowground pools in open pasturelands (OP), fodder banks (FB), live fences (LF) dispersed trees on pasturelands (DT), secondary forests (SF), and primary forests (PF) following a gradient of land-use in Jalisco, Chiapas, and Campeche, Mexico (the definitions and characteristics of sampled land-uses are presented in Fig. 1). We also explored the relationships between carbon storage, land-use history, and soil properties that can explain SOC variations in these land-uses. We hypothesized that C storage in biomass and soil increases with the gradient of land-use from ecologically simple open pasturelands to more diverse, ecologically complex SPS, secondary and primary forests.

## Results

### Tree species richness and diversity

Tree species richness was higher in PF, followed by SF, DT, LF, and FB. OP have no trees, so tree richness values for this land-use is zero. The Sorenson´s similarity coefficient, which is a measure of the degree of similarity with our reference land-use (PF), ranged from 0.00 to 1.00, where 1.00 indicates that all the species are common between PF and the respective land-use and 0 indicates that no species are common (Table 1). The Shannon–Weaver biodiversity index ranged from 0 to 3.62, the lowest values corresponded to OP and the highest to the PF of the Campeche region. The highest values of the Shannon–Weaver (H´) index indicate a greater diversity of tree species. The general order of tree species diversity values was: OP < FB < LF < DT < SF < PF, except in Chiapas where PF showed a lower diversity index than SF and two of our silvopastoral systems (DT and LF). The Pilou´s evenness index (J) varied according to the region and land-uses. The J index can oscillate between 0.0 and 1.0, where the first indicates completely unequal while the last indicates total equality of abundance. The evenness index of primary forests in the Chiapas region was lower than other land-uses, indicating that a few species were more abundant than others (Table 1).

**Table 1:** Tree species richness, Sorenson´s similarity coefficients with primary forest species, Shannon´s diversity index, and Pilou´s evenness indices between land-uses for three study regions.

Land-use (from simplified to complex)	Tree species richness, N (Sorenson’s similarity coefficient, CC)			Shannon’s diversity index, H (Pilou’s evenness index, J)		
	Jalisco	Campeche	Chiapas	Jalisco	Campeche	Chiapas
Open pasturelands	0 (0.00)	0 (0.00)	0 (0.00)	0 (0.00)	0 (0.00)	0 (0.00)
Fodder banks	1 (0.00)	nd	2 (0.00)	0 (0.00)	nd	0.53 (0.77)
Live fences	nd	11 (0.16)	16 (0.19)	nd	1.44 (0.60)	2.20 (0.79)
Dispersed trees	13 (0.42)	11 (0.26)	16 (0.37)	2.09 (0.82)	1.56 (0.65)	2.29 (0.83)
Secondary forest	20 (1.00)	35 (0.54)	25 (0.27)	2.37 (0.79)	2.47 (0.69)	2.46 (0.77)
Primary forests	nd	65 (1.00)	27 (1.00)	nd	3.62 (0.86)	1.80 (0.55)

*PF* Primary forests, *SF* Secondary forests, *DT* Dispersed tree in pasturelands, *LF* Live fences, *FB* Fodder banks, *OP* Open pasturelands.

*nd* data not available.

### Living tree biomass carbon stocks

The mean C stocks in tree AGB varied between 1.8 and 134.6 Mg C ha^−1^ with significant differences between land-uses, the lowest value corresponded to FB of Chiapas and the highest to PF of the same region (Table 2). Woody biomass was absent in OP due to the absence of trees. The AGB stock differences between study sites were not significant for FB, LF, DT, and PF. Only in SF, Campeche, and Chiapas sites stored higher C stocks compared to Jalisco. DT stored 12.6 Mg C ha^−1^ in Chiapas sites, 14.8 Mg C ha^−1^ in Jalisco, and 34.8 Mg C ha^−1^ Campeche with no significant differences between them. Average among three regions, DT stored 18.2 Mg C ha^−1^ in AGB and was significantly higher than LF (2.6 Mg C ha^−1^) and FB (2.2 Mg C ha^−1^) but did not differ statistically from SF (25.0 Mg C ha^−1^). Carbon stocks in root biomass ranged from 0.5 to 31.6 Mg C ha^−1^ and followed a similar pattern with AGB (Table 2). Averaged among regions, DT stored about 15% of PF biomass and 73% of SF tree biomass C stocks.

**Table 2:** Back-transformed weighted means and respective 95% confidence intervals of carbon stocks in above-ground biomass (AGB), root biomass (RB), and total tree biomass (Mg C ha^−1^) between different land-uses in three regions of Mexico. Tree total biomass: AGB + RB. Superscript uppercase letters indicate significant differences between land-uses, while lowercase letters indicate statistical differences between geographical regions (Tukey HSD, p < 0.05).

Land-use	Region	AGB (Mg C ha^−1^)			RB (Mg C ha^−1^)			Tree total (Mg C ha^−1^)		
		Mean	CI − 95%	CI + 95%	Mean	CI − 95%	CI + 95%	Mean	CI − 95%	CI + 95%
Fodder banks	Jalisco	3.0^a^	0.5	17.1	0.9^a^	0.2	4.7	4.0^a^	0.7	21.7
	Campeche	nd	nd	nd	nd	nd	nd	nd	nd	nd
	Chiapas	1.8^a^	0.7	3.4	0.5^a^	0.2	1.1	2.2^a^	0.9	4.5
	All regions	2.2^A^	1.1	3.8	0.7^A^	0.4	1.1	2.9^A^	1.5	4.9
Live fences	Jalisco	nd	nd	nd	nd	nd	nd	nd	nd	nd
	Campeche	2.1^a^	0.5	9.1	0.7^a^	0.2	2.6	2.7^a^	0.6	11.7
	Chiapas	3.1^a^	1.9	5.1	1.0^a^	0.6	1.5	4.1^a^	2.5	6.6
	All regions	2.6^A^	1.4	4.6	0.8^A^	0.5	1.4	3.4^A^	1.9	6
Dispersed trees	Jalisco	14.8^b^	9.2	23.8	4.1^b^	2.6	6.4	18.9^b^	11.8	30.2
	Campeche	34.8^b^	14.5	83.3	9.0^b^	4	20.3	43.9^b^	18.6	103.6
	Chiapas	12.6^b^	5.7	27.7	3.5^b^	1.7	7.3	16.1^b^	7.4	35
	All regions	18.2^B^	12.2	27.2	5.0^B^	3.4	7.2	23.2^B^	15.6	34.4
Secondary forests	Jalisco	9.3^a^	5.5	15.7	2.7^a^	1.6	4.3	11.9^a^	7.1	20
	Campeche	55.0^b^	40.4	74.9	13.8^b^	10.4	18.4	68.8^b^	50.8	93.3
	Chiapas	31.3^b^	16.4	59.8	8.2^b^	4.5	14.9	39.5^b^	20.9	74.7
	All regions	25.0^B^	16.2	38.8	6.7^B^	4.4	10	31.7^B^	20.6	48.8
Primary forests	Jalisco	nd	nd	nd	nd	nd	nd	nd	nd	nd
	Campeche	110.8^c^	56.9	215.6	26.4^c^	14.3	49	137.2^c^	71.2	264.5
	Chiapas	134.6^c^	99.6	181.8	31.6^c^	23.9	41.8	166.2^c^	123.5	223.6
	All regions	127.3^C^	100.2	161.7	30.0^C^	24.1	37.5	157.3^C^	124.3	199.2

*nd* data not available.

### Aboveground grass biomass, surface litter, and deadwood carbon stocks

**Figure 2:**
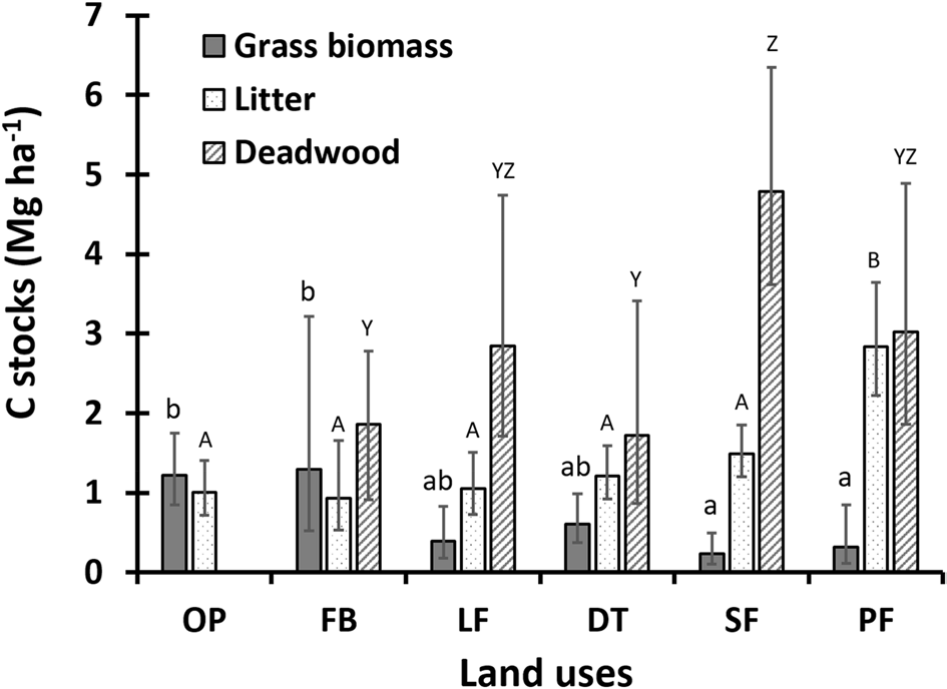
Average carbon stocks in grass biomass, ground litter, and deadwood material in different land-uses. OP = Open pastures, FB = Fodder banks, LF = Live fences, DT = Pasturelands with dispersed trees, SF = Secondary forests, PF = Primary forests. Error bars indicate the respective 95% confidence intervals. Different letters above the bars indicate significant differences between land-uses (p < 0.05).

Land-uses differ significantly in the amount of C stored in aboveground grass biomass (Fig. 2). Back-transformed mean C stock in this pool ranged from 0.24 to 1.29 Mg C ha^−1^. In this pool, FB and OP hold the higher C stock compared to SF and PF. Mean C stock in surface litter pool ranged from 0.93 to 2.84 Mg C ha^−1^, PF stored significantly higher C stock compared to other land-uses (Fig. 2). Regarding C stocks in the deadwood pool, SF (4.79 Mg C ha^−1^) stored significantly higher than FB and DT but not statistically different from PF (3.02 Mg C ha^−1^) and LF (2.85 Mg C ha^−1^).

### Soil organic carbon stocks

The SOC was the biggest C pool across these land-uses, demonstrating the importance of conserving this C reservoir. The SOC stocks (0–30 cm) showed a differential response to land-uses according to the region of the study. In Jalisco region, SOC stocks varied from 130.3 to 145.9 Mg C ha^−1^, the lower value corresponded to OP and the upper value to SF. However, differences between land-uses were not significant (Table 3). In Campeche region, SOC stocks ranged between 108.7 and 152.5 Mg C ha^−1^, the lower value also corresponded to OP and the upper value to DT, with statistically significant differences. In Chiapas region, SOC stocks ranged from 152.5 to 227.5 Mg C ha^−1^, the lower value corresponded again to OP and the upper value to LF, with statistically significant differences (Table 3).

**Table 3:** Average soil organic carbon (SOC) stocks (Mg C ha^−1^) to 30 cm depth by land-uses across regions.

Land-use	Region	SOC stocks to 30 cm depth (Mg C ha^−1^)		
		Mean	Lower 95% CI	Upper 95% CI
Open pasturelands	Jalisco	130.3^x^	115.3	145.3
	Campeche	108.7^a^	92.3	125.1
	Chiapas	152.3^p^	137.4	167.3
Fodder banks	Jalisco	145.7^x^	124.5	166.9
	Campeche	nd	nd	nd
	Chiapas	175.5^p^	160.5	190.5
Live fences	Jalisco	nd	nd	nd
	Campeche	126.6^ab^	110.1	143.0
	Chiapas	227.5^q^	212.5	242.5
Dispersed trees on pasturelands	Jalisco	144.3^x^	127.9	160.7
	Campeche	152.3^b^	135.9	168.7
	Chiapas	182.5^pq^	167.6	197.5
Secondary forests	Jalisco	145.9^x^	130.9	160.9
	Campeche	127.7^ab^	111.3	144.1
	Chiapas	186.8^pq^	165.6	208.0
Primary forests	Jalisco	nd	nd	nd
	Campeche	129.0^ab^	112.6	145.4
	Chiapas	220.5^q^	204.1	236.9

Different letters indicate statistical differences between land-uses within the same region (Tukey HSD, p < 0.05).

nd: data not available.

### Soil organic carbon concentration

**Figure 3:**
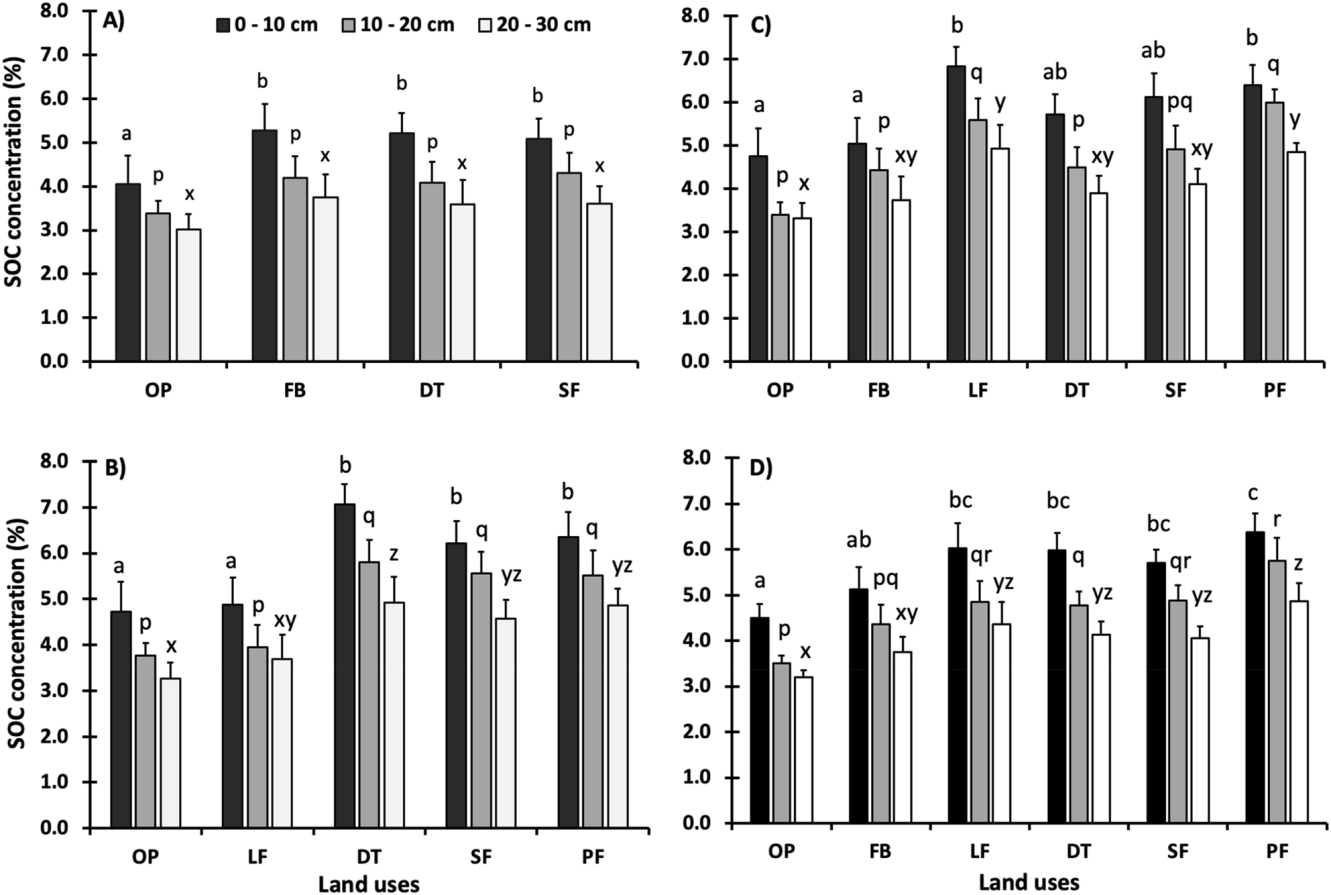
Soil organic carbon (SOC) content (%) across sampled land uses by soil depth category. (**A**) Jalisco, (**B**) Campeche, (**C**) Chiapas, (**D**) All regions. Lowercase letters above the bars indicate significant differences between land-uses in the same soil depth category. The y-axis shows SOC content (%) and the x-axis shows the different land-uses sampled, where OP = Open pasturelands, FB = Fodder banks, DT = Pasturelands with dispersed trees, LF = Live fences, SF = Secondary forests, PF = Primary forests.

The SOC concentration (%) varied between land-uses and soil depth categories (p < 0.05) but without significant interactions between them in each region. In the Jalisco region, SOC concentrations in 0–10 cm depth, varied from 4.1 to 5.5%, the lower value corresponded to OP and the upper value to DT, with statistically significant differences. At 20–30 cm soil layer, it ranged from 3.2 to 3.8%, but no statistical differences were observed among land-uses (Fig. 3A). In the Campeche region, SOC content at 0–10 cm layer varied from 4.9 to 7.3%, the lower value corresponded to LF and the upper value to DT. A similar trend was observed at 10–20 cm depth. At 20–30 cm layer, it varied between 3.7 and 5.0%, where the lower value corresponded to OP and the higher value to SF (Fig. 3B). In the Chiapas region, SOC concentration at 0–10 cm layer ranged from 4.7 to 6.8%, the lower value corresponded to OP and the upper value to LF. At 10–20 cm layer, it differed from 3.4 to 6.0%, with the lowest value from OP and the highest from PF. At 20–30 cm layer, it ranged from 3.3 to 4.9%, with OP being the smallest (Fig. 3C). When averaged among regions, LF, DT, and SF SOC contents did not differ statistically from PF at 0–10 cm and 20–30 cm layers (Fig. 3D).

### Fine root biomass (FRB)

The effect of land-uses on the average FRB varied by region. In the Jalisco region, FRB at 0 – 30 cm depth ranged from 2.10 to 5.80 Mg ha^−1^, where OP showed the smallest amount and SF the highest. In the Campeche region, FRB ranged from 3.04 to 9.82 Mg ha^−1^, where the smallest value corresponded to LF and the greatest to PF (Table 4). In the Chiapas region, FRB to 30 cm depth varied from 2.71 to 12.50 Mg ha^−1^, the smallest being the FB and the greatest the LF. Fine root biomass in OP, FB, and DT did not differ statistically (Table 4).

**Table 4:** Fine root biomass (Mg ha^−1^) in the soils across different land-uses by depth classes and region.

Land-use/soil depth	Fine root biomass stocks, Mg ha^−1^, mean (± 95% CI)		
	Jalisco	Campeche	Chiapas
**Open pasturelands**			
0–10 cm	1.28 (± 0.70)^a^	3.00 (± 1.52)^ab^	2.99 (± 1.27)^a^
10–20 cm	0.41 (0.25)^p^	1.41 (± 1.03)^p^	0.54 (± 0.23)^p^
20–30 cm	0.41 (± 0.28)^x^	0.25 (± 0.15)^x^	0.24 (± 0.07)^x^
Σ(0–30) cm	2.10 (± 0.92)^A^	4.66 (± 1.99)^A^	3.77 (± 1.35)^A^
**Fodder banks**			
0–10 cm	2.26 (± 1.11)^a^	nd	1.66 (± 0.45)^a^
10–20 cm	0.95 (± 0.70)^p^	nd	0.75 (± 0.31)^p^
20–30 cm	0.68 (± 0.50)^x^	nd	0.29 (± 0.09)^x^
Σ(0 – 30) cm	3.90 (± 1.92)^AB^	nd	2.71 (± 0.64)^A^
**Live fences**			
0–10 cm	nd	2.09 (± 1.08)^a^	4.91 (± 2.41)^a^
10–20 cm	nd	0.57 (± 0.18)^p^	4.87 (± 3.66)^p^
20–30 cm	nd	0.38 (± 0.16)^x^	2.72 (± 3.51)^x^
Σ(0–30) cm	nd	3.04 (± 1.13)^A^	12.50 (± 7.74)^B^
**Dispersed trees on pasturelands**			
0–10 cm	2.19 (± 1.96)^a^	1.71 (± 0.78)^a^	1.28 (± 0.47)^a^
10–20 cm	1.07 (± 0.90)^p^	1.10 (± 0.65)^p^	1.40 (± 0.70)^p^
20–30 cm	0.72 (± 0.51)^x^	1.11 (± 0.67)^xy^	0.96 (± 0.96)^x^
Σ(0–30) cm	3.98 (± 2.46)^AB^	3.92 (± 1.12)^A^	3.64 (± 1.51)^A^
**Secondary forests**			
0–10 cm	1.79 (± 0.65)^a^	2.72 (± 1.69)^ab^	1.68 (± 1.21)^a^
10–20 cm	2.20 (± 1.49)^p^	1.88 (± 0.87)^p^	1.97 (± 1.30)^p^
20–30 cm	2.48 (± 1.59)^x^	1.73 (± 0.92)^xy^	1.99 (± 1.44)^x^
Σ(0–30) cm	5.80 (± 2.32)^B^	6.33 (± 2.65)^AB^	6.64 (± 2.08)^AB^
**Primary forests**			
0–10 cm	nd	4.88 (± 1.63)^b^	5.21 (± 3.00)^a^
10–20 cm	nd	2.72 (± 1.48)^p^	2.69 (± 0.99)^p^
20–30 cm	nd	2.22 (± 1.01)^y^	4.25 (± 2.93)^x^
Σ(0–30) cm	nd	9.82 (± 1.96)^B^	12.15 (± 4.44)^B^

Different letters indicate statistical differences between land-uses (p < 0.05) within the same depth class. CI = Confidence intervals.

nd: data not available.

### Soil bulk density

The average soil bulk density (BD) tends to be higher in OP compared to other land-uses, but this tendency differed by region. In the Jalisco region, BD ranged between 1.1 and 1.3 g cm^−3^ with OP being significantly higher than other land-uses. In the Campeche region, it ranged between 0.8 and 1.0 g cm^−3^, with PF and SF showing the lowest values. In the Chiapas region, BD ranged from 1.2 to 1.4 g cm^−3^, with OP presenting the greatest value (Table 5).

**Table 5:** Soil bulk density (g cm^−3^) across land-uses by depth category and study region.

Land-use	Soil depth	Soil bulk density, g cm^−3^, mean (± 95% CI)		
		Jalisco	Campeche	Chiapas
**Open pasturelands**				
	0–10 cm	1.2 (± 0.07)^a^	0.9 (± 0.10)^ab^	1.3 (± 0.06)^a^
	10–20 cm	1.3 (± 0.08)^p^	0.9 (± 0.14)^pq^	1.4 (± 0.04)^q^
	20–30 cm	1.3 (± 0.08)^y^	1.0 (± 0.11)^x^	1.4 (± 0.04)^x^
	0–30 cm	1.3 (± 0.05)^B^	1.0 (± 0.07)^AB^	1.4 (± 0.03)^B^
**Fodder banks**				
	0–10 cm	1.1 (± 0.08)^a^	nd	1.3 (± 0.05)^a^
	10–20 cm	1.1 (± 0.04)^p^	nd	1.3 (± 0.05)^pq^
	20–30 cm	1.1 (± 0.09)^xy^	nd	1.4 (± 0.04)^x^
	0–30 cm	1.1 (± 0.04)^A^	nd	1.3 (± 0.03)^AB^
**Live fences**				
	0–10 cm	nd	1.0 (± 0.10)^b^	1.3 (± 0.05)^a^
	10–20 cm	nd	1.1 (± 0.10)^q^	1.3 (± 0.07)^pq^
	20–30 cm	nd	0.9 (c 0.12)^x^	1.3 (± 0.06)^x^
	0–30 cm	nd	1.0 (± 0.06)^B^	1.3 (± 0.03)^AB^
**Dispersed trees on pasturelands**				
	0–10 cm	1.1 (± 0.10)^a^	0.9 (± 0.09)^a^	1.3 (± 0.06)^a^
	10–20 cm	1.2 (± 0.10)^p^	0.8 (± 0.09)^p^	1.3(± 0.05)^pq^
	20–30 cm	1.2 (± 0.06)^xy^	0.9 (± 0.07)^x^	1.3(± 0.06)^x^
	0–30 cm	1.1 (± 0.05)^A^	0.9 (± 0.05)^A^	1.3 (± 0.03)^AB^
**Secondary forests**				
	0–10 cm	1.1 (± 0.07)^a^	0.7 (± 0.12)^a^	1.2 (± 0.05)^a^
	10–20 cm	1.2 (± 0.08)^p^	0.8 (± 0.12)^p^	1.2 (± 0.09)^p^
	20–30 cm	1.1 (± 0.12)^x^	0.8 (± 0.09)^x^	1.3 (± 0.07)^x^
	0–30 cm	1.1 (± 0.05)^A^	0.8 (± 0.06)^A^	1.2 (± 0.04)^A^
**Primary forests**				
	0–10 cm	nd	0.8 (± 0.14)^a^	1.2 (± 0.05)^a^
	10–20 cm	nd	0.8 (± 0.10)^p^	1.3 (± 0.07)^pq^
	20–30 cm	nd	0.8 (± 0.13)^x^	1.3 (± 0.05)^x^
	0–30 cm	nd	0.8 (± 0.07)^A^	1.3 (± 0.06)^AB^

Different letters indicate the statistical differences between land-uses (p < 0.05). CI = Confidence intervals.

nd: data not available.

### Relationship between variables of land-use history, soil properties, and carbon storage

**Figure 4:**
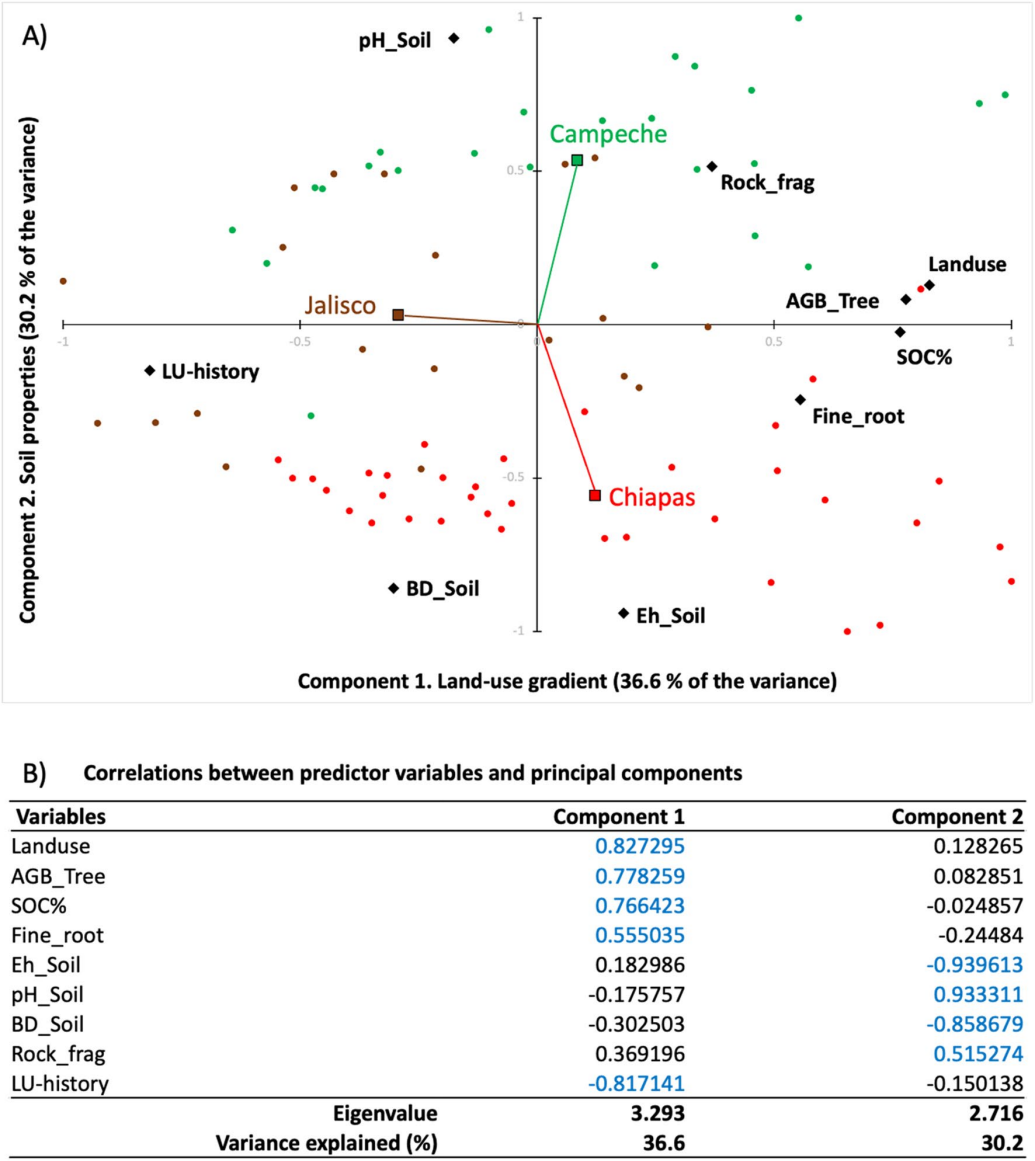
Component plotting of multiple variables including soil organic carbon content (%) based on the principal component analysis (PCA). (**A**) PCA plot with observations; (**B**) Correlations between principal components and predictor variables. AGB = aboveground biomass, LU = land use, BD = soil bulk density (g cm^−3^), Eh = soil oxidation–reduction potential (ORP, mV), SOC = soil organic carbon concentrations (%).

We observed that tree aboveground biomass, fine root biomass, and SOC concentrations were positively related variables, indicating that the incorporation of trees enhances SOC storage (Supplementary Table T1). The relationships among variables such as tree biomass, fine root biomass, SOC concentration (%), soil pH, oxidation–reduction potential (Eh), bulk density, land-use history (years after forest conversion), and presence of rock fragments are shown in a principal component (PC) analysis (Fig. 4). The first two principal components with eigenvalues > 1 explained 66.8% of the variance (Fig. 4A). We observed that land use-gradient, land-use history, tree biomass, SOC concentrations, and fine root biomass were correlated more to PC1. Hence, we named this component as land-use gradient. We named the component 2 as soil properties because soil bulk density, pH, redox potential (Eh), and rock contents correlated more to this PC. In the biplot, we observe that tree biomass, SOC concentrations, and fine root biomass were clustered horizontally while soil pH, Eh and bulk density clustered vertically. Carbon storage was inversely related to land-use history (years that the land was used for animal grazing). Observations of Campeche region were distributed more to upper part, characterized by the higher soil pH but diverse in SOC concentrations. Jalisco region was characterized by lower SOC concentration and the longer history of land use. Chiapas region was characterized by the lower soil pH but showed a wider range in SOC concentrations (Fig. 4).

## Discussion

Carbon sequestration through SPS can contribute significantly to mitigating greenhouse gas emissions from the livestock sector and reduce the environmental footprints of animal products^37,40,62^. Avoided conversion of forests to OP leads to higher C retention within the landscape, while the transformation of OP to SPS enhances C accrual from the atmosphere^46^. In addition to C stored in biomass, these systems contribute to avoiding the loss of SOC due to the possible conversion to OP systems. In this study, FB, LF, DT, SF, and PF stored 26.5, 39.8, 45.3, 54.2, and 163.1% higher total C stocks respectively compared to OP, highlighting the importance of SPS and forests in livestock-dominated landscapes. The size of different C pools differs across land-uses because of the functional, structural, and compositional differences^11,72–74^. Silvopastoral systems (SPS) and forests have more vertical layers due to the presence of trees, are richer in species diversity, and have deeper root systems than OP^32,65,75–77^. The amount of living biomass stocks across the different land-uses varied because of the tree density, vegetation structure, and species differences, between land-uses^16,78^.

For example, in the LF, trees are planted in rows on the borders of about one-ha plots, and the interior matrix of the land is covered with grasses (i.e., when separating paddocks)^79^. Hence, the total tree density per ha is lower in this system compared to DT and forests (PF and SF), which explains the lower carbon stocks in living tree biomass^80^. Besides, in some cases, trees are pruned once a year to provide animal fodder and maintain the accessible height of the fences. In contrast, in the DT land-use, trees are found scattered within the pastureland matrices without any strict distance or row arrangements. Such a random distribution leads to higher access to sunlight resulting in wider crown diameter and bigger trees^81^. The average tree basal area (ha^−1^) was also higher in DT compared to LF, which increased the living tree biomass stocks^71^. Some of the trees in the DT are remnants of the original PF left uncut during the land conversion which are relatively bigger than other trees grown after. Tree density is even higher in SF, but the average tree size is relatively smaller if compared to PF. Across the study areas, PFs are generally composed of bigger native trees with higher stand-level wood density compared to SF, resulting in higher carbon stocks in woody biomass^82^.

Another factor that affects C storage is the intensity of land-use and management. Open pasturelands and other SPS (LF and DT) were grazed continuously with the same intensity but the presence of trees and their arrangements make them different in terms of biomass growth and organic matter input to the system^80,83,84^. Fodder banks are harvested frequently to feed animals, but grazing is limited in these systems^59,85^. Secondary forests are occasionally grazed but with lower intensity where biomass removal is lower compared to OP and SPS. Biomass removal by cattle grazing in PF is very rare making it a low-intensity system. Such differences in grazing management partly explain the change in above- and belowground organic matter input leading to the differences in total carbon storage between land-uses within the same landscape^57,86,87^. Reduced organic matter input, C loss from soil erosion, continuous loss from heterotrophic soil respiration, and reduced soil biological activity for organic matter stabilization are some of the principal causes of SOC depletion in OP^44,73,88,89^.

SOC stocks (Mg ha^−1^) are principally affected by SOC concentrations (%) but also by soil bulk density. Higher SOC concentrations explain the increased SOC stocks in forests (PF and SF) and SPS but the higher. Soil bulk density leads to higher soil mass per hectare and apparently higher SOC stocks. However, SOC concentrations (%) were significantly lower in OP compared to SPS or forest land-uses (PF and SF).

Forests (PF and SF) and SPS, are generally composed of plant species with diverse rooting systems that promote the accumulation of root-derived organic matter and microbial detritus to deeper soils^90,91^. In Alberta Canada, SPS had 30% higher SOC compared to open herb systems^92^. Higher carbon sequestration in SPS is also reported in Latin America and other parts of the world^64,93,94^. In addition to the higher accumulation of organic matter, the loss of carbon by mineralization and heterotrophic respiration is lower in SPS due to micro-climatic buffering^48,69,95^. The integration of trees on pasturelands improves soil microbial and enzymatic activity with the potential to increase organic carbon storage^36,96,97^.

The principal component analysis showed that land-use history (the time after forest conversion, years) was another important variable and related inversely with SOC %, but positively to soil bulk density (Fig. 4). Such a relationship indicates that the longer the land is used for livestock farming, the lower the concentration of SOC. The positive relations between land-use history and soil bulk density indicate that soil compaction is increasing with the time that the land is used for livestock farming (Fig. 4). However, further studies are required to have a deeper understanding of the role of land-use history on SOC sequestration in these areas. The soils of the Campeche region are calcareous with high Ca and Mg cations with pH slightly higher than neutral due to the karstic parent material^98^. Independent of land-use, SOC concentrations % (but not SOC stocks) were slightly higher in the Campeche region. Among others, the sorption of negatively charged organic matter by cation bridging in Ca-rich soils could have increased the stabilization of SOC, leading to higher C accumulation^91,97,99^. However, the higher presence of rocks in the soil of this region reduced the SOC stocks (Mg ha^−1^) because of the lower soil mass equivalent.

Storing PF level C in grazing lands should be an ambitious goal because it can significantly lower grass availability and hence reduce livestock productivity. However, increasing C stocks to the level of LF and DT can be considered viable options. Evidence showed that better management of pasturelands by incorporating trees should increase animal production intensity by restoring soil fertility allowing farmers to spare areas for conservation and hence higher C storage at the landscape scale^12,100–102^. Besides, conserving forest fragments or increasing trees within the matrix of livestock-dominated landscapes is a dual strategy that not only helps C sequestration but also benefits biodiversity conservation, heat stress management, dry season feeding, and overall ecosystem resilience^35,36,103^. Conserving even a small area of PF can help farmers as a seed pool for the regeneration of native trees on the surrounding landscape^104^.

Integrating different silvopastoral and forested land-uses within the farmlands can be recommended as a good practice that promotes the synergies between climate change adaptation and mitigation benefits. This is especially important in the hotspots where livestock farming is often considered to be a threat to important natural protected areas such as La Sepultura Biosphere Reserve in Chiapas, Calakmul, and Balam Ku Biosphere Reserves in Campeche, and Chamela-Cuixmala and Sierra de Manantlán Biosphere Reserves in Jalisco.

Mexico in its Intended Nationally Determined Contributions (INDC) has proposed to reduce total GHG emissions by 22% and achieve zero deforestation by 2030^105^. It is also stipulated that net emissions from land-use change and the forestry sector would go negative (i.e., higher carbon capture than emissions). This means the Agriculture Forestry and Other Land use (AFOLU) sector should mitigate 53 Tg CO_2_e per year (7 Tg from agriculture + 46 Tg from land-use change) by 2030 as compared to the business-as-usual scenario^105^. However, land-use change from forestry to pastureland is still common because of the increasing interest in livestock farming^106^. As a consequence, strategic frameworks and actions are still not well articulated to promote SPS as a carbon mitigation strategy at the local level, even though there´s plenty of evidence of its potential. Currently, 80.3 million ha of land is used for livestock grazing in Mexico, most of them as OP^17^. If 25% of the current OP could be converted to SPS, reaching the total C stock level to DT or LF found in this study, an additional 815 Tg C could be captured in these lands. It would take 25–80 years to sequester this amount of C, taking into account the variation in C sequestration rates of SPS (0.5–1.8 Mg C ha^−1^ year^−1^) in the region^80^. Chiapas currently has about 3.79 million hectares of grasslands^107^. Considering that there would be no deforestation for extra grassland expansion, a 25% SPS adoption could mitigate 63% of the projected livestock sector GHG (3.41 Tg CO_2_e) by 2050 in Chiapas. Campeche currently has about 1.05 million hectares of grasslands^107^. Considering the scenario that there would be no further deforestation for grassland expansion, 25% adoption of SPS could mitigate 58% of the projected livestock sector GHG (1.01 Tg CO_2_e) by 2050 in Campeche. Jalisco currently has 1.11 million hectares of grasslands^107^. With the 25% SPS adoption, only 8% of the projected 2050 livestock sector GHG emissions (8.55 Tg CO_2_e) could be mitigated in Jalisco. This is attributed to the fact that livestock production in Jalisco is more intensive than in Chiapas and Campeche region, which requires additional technological measures to reduce livestock sector GHG.

At the farm level, restoring 25% of the degraded pasturelands to achieve C stock level to SPS and/or conserving high C density forest remnants may require good technical guidance and incentives. Such a change in land-use and management could contribute significantly to meeting the ambitious national goals of Mexico regarding mitigation and adaptation to climate change. However, the institutional framework, farmers´ capacity to adopt SPS, and the time required to sequester this amount of carbon should be evaluated. It requires multiple actions to strengthen farmers´ capacity and incentivize carbon resilient land-use practices for socio-economic benefits at the local level^108^. Avoided deforestation and inclusion of trees as SPS on livestock farmlands would mitigate net carbon emissions and achieve carbon–neutral livestock production^109^. On the global scale, significant carbon benefit can be obtained by converting pasture monocultures (OP) to SPS because a large extension (~ 3.3 billion hectares) of land is currently used for livestock grazing^17,101^.

Looking at the other perspective, the continuous reduction of SOC and soil fertility in OP can reduce grass productivity. As a consequence, farmers can opt to convert the adjacent PF to increase the area of pastureland. Unless intervened technically and institutionally, there exists a great danger of losing high carbon density ecosystems (like PF = 349.2 Mg C ha^−1^) to establish low carbon density ones (OP = 132.7 Mg C ha^−1^). From this study, we came to know that none of the current SPS evaluated, i.e., FB (167.9 Mg C ha^−1^), LF (185.5 Mg C ha^−1^), or DT (192.9 Mg C ha^−1^), can store the amount of C accumulated by PF (349.2 Mg C ha^−1^), being the main differences driven by the trees present in PF (Supplementary Fig. F1). Crop and grassland expansion in southern Mexico accelerated during the 60 s to 90 s when federal and local governments incentivized forest conversion to OP and croplands^22,110^. Farmers during that period were trained to maintain their grasslands without trees, looking like a snooker board. In recent decades, the most common trend is the conversion of croplands, especially maize + bean, to grasslands because of higher income from animal farming^111^. Longer-duration and frequent droughts in northern arid parts of Mexico have also triggered recent livestock expansion in the south-southwest of the country. In this context, we estimate that more than 90% of the current SPS in the region were derived from the inclusion (planting or natural regeneration) of trees in OP. Therefore, the major part of the differences in C stock between SPS and OP is considered as the true offset of emissions.

Efficient strategies are needed at the local and regional levels to protect such vulnerable carbon stocks by conserving high C density systems and increasing carbon stocks in the low C density ecosystems^112^. It is necessary to promote the intensification of livestock production through SPS and other good management practices to take better advantage of biomass for animal production and C storage in the deforestation and grassland expansion hotspots. The development of evidence-based mechanisms for higher production per unit area would compensate for the opportunity cost of further land conversion and promote the release of land for restoration to high C density ecosystems in these livestock-dominated landscapes.

These results have implications for designing and scaling up the adoption of climate-smart livestock farming systems. We highlight the importance of conserving remnant PF, restoring degraded OP through the establishment of SPS, and land sparing for the increase of SF. We showed that SPS enhances C storage contributing to mitigating GHG emissions from livestock farming, in addition to species and structural diversification. Knowledge of the region- and system-specific variations on C storage help identify appropriate SPS according to the soil, climate, and landscape characteristics. Furthermore, we highlighted the relationship between above and belowground C pools, soil properties, and land-use history to be considered while implementing and measuring the contribution of C-enhancement strategies in livestock-dominated landscapes. Our results can partly contribute to decisions regarding the inclusion of SPS in the development of Nationally Appropriate Mitigation Actions (NAMA) for the livestock sector in Mexico and other countries of similar contexts.

## Materials and methods

### Study sites

**Figure 5:**
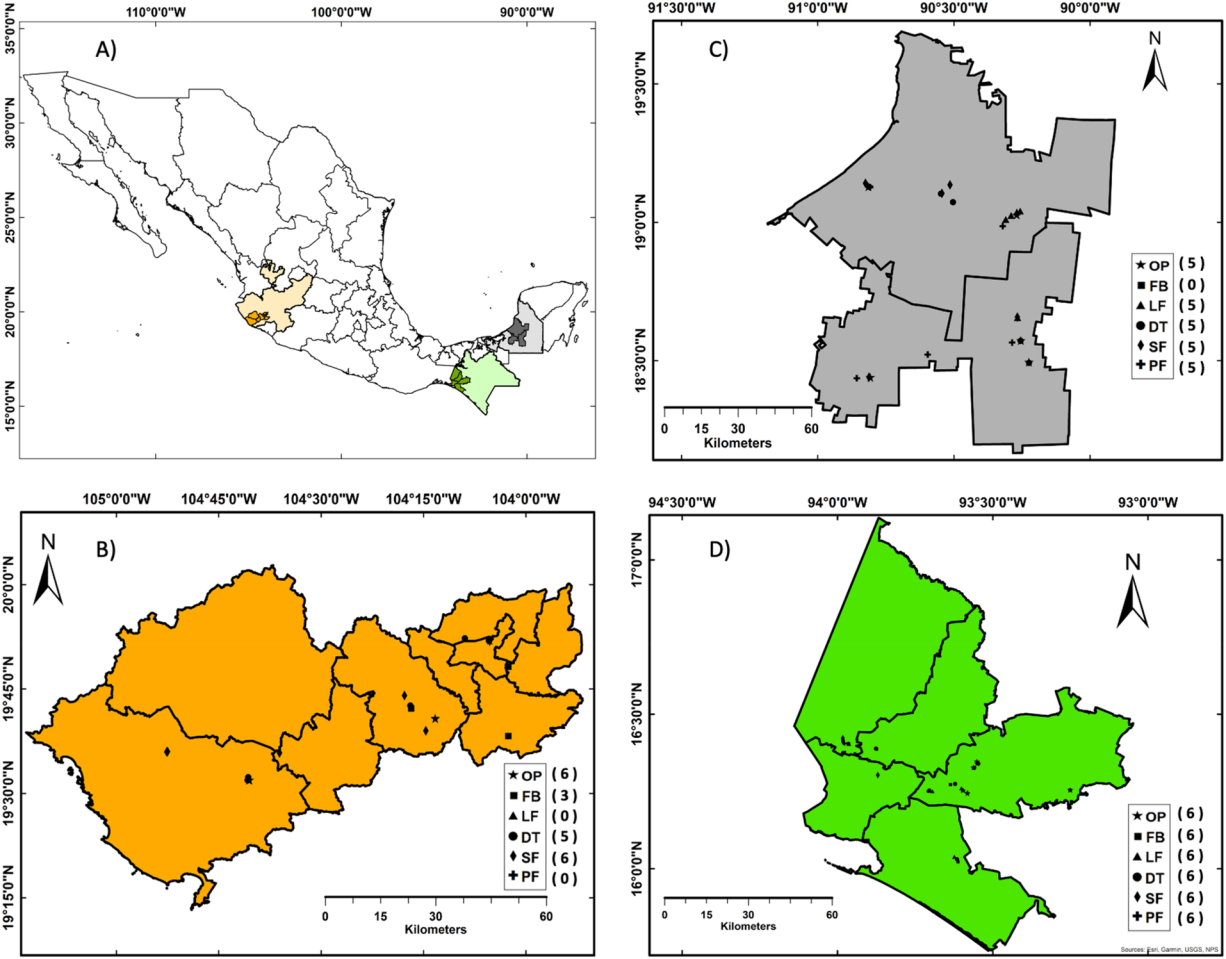
Study area location and distribution of sampling plots across landscapes. (**A**) States (light color) and study areas (darker color) where sampling was conducted. The letters and symbols correspond to the different types of land-uses, OP = Open pasturelands, FB = Fodder banks, LF = Live fences, DT = Pasturelands with dispersed trees, SF = Secondary forests, and PF = Primary forests. (**B**–**D**) Close-ups of the study sites in Jalisco (**B**), Campeche (**C**), and Chiapas (**D**). The values in the parenthesis show the number of plots sampled (n) for each land-use.

The study was carried out in three tropical livestock-dominated regions of Mexico: (1) Pacific coastal watershed in Jalisco, (2) La Sierra Madre in Chiapas, and (3) Southern Yucatan Peninsula in Campeche (Fig. 5). These regions have different soil pH varying between 6.0 and 7.0 in Jalisco, 4.5 and 5.5 in Chiapas, and 7.0 and 7.5 in Campeche, important biophysical characteristics of all regions are presented in Supplementary Table T2. Selected study regions contain important natural reserves and habitat conservation areas, some of them being part of the large Mesoamerican Biological Corridor initiative. However, the pressure on natural resources is increasing due to natural and anthropogenic factors^113^. Furthermore, these regions are prioritized for REDD + (reduction of emissions from deforestation and degradation + C enhancement) early actions in Mexico. The selected study sites correspond to areas of intervention of the project “Scaling up biodiversity conservation through climate-smart agrosilvopastoral practices in landscapes dominated by cattle-raising systems in three regions of Mexico”^114^.

We selected the most common land-uses from each region where bovine cattle grazing is the principal farming activity. We conducted farmers´ interviews to learn about land-use history, stocking rates, and land management practices of each study site. Based on tree richness and diversity, vertical structure, and tree cover we identified a land-use gradient from simple to ecologically complex^115^ which is reflected as follows considering the six studied land-uses: open pasturelands (OP), fodder banks (FB), live fences (LF), pasturelands with dispersed trees (DT), secondary forests (SF), and primary forests (PF) (Fig. 5). Conventional OP was considered the simplest form of land-use due to the lack of trees, followed by FB which included one or two tree species, LF including multiple tree species, DP including multiple tree species, and greater tree cover (compared to previous land-uses), SF that share multiple native tree species with primary forests, and finally, PF (old-growth) which was considered the more complex form and was also used as a reference land-use^115^. We sampled a total of 79 plots across the three study areas (Fig. 5 shows the number of plots by land-use from each region). Sampled SF was occasionally used for livestock grazing, particularly during the dry season while PF was not grazed.

### Measurement of carbon stocks in different pools

#### Tree biomass and diversity

We first delimited the sampling plots or fence distance to measure all the trees of ≥ 2.5 cm diameter at breast height (DBH). The size of each sample plot to measure tree biomass was 1000 m^2^ (40 m × 25 m) for FB, DT, SF, and PF. For LF, we measured the trees within a linear distance of 100 m. Since trees were absent in OP, we established similar plots for the nested sampling of other C pools.

We measured tree DBH (at 1.3 m from the ground level) by using diameter tapes and total height with the trigonometric approach using clinometers. All trees found within the plots were identified to the species level allotting the respective local and scientific names. We searched for wood density data in the global wood density database and other published literature^116^ and quantified the aboveground biomass (AGB) by using an allometric equation for tropical trees^117^.

$$AGB={0.0673 \left(\rho {D}^{2}H\right)}^{0.976}$$

where AGB is the aboveground biomass of the tree (kg), $\rho$ is the wood density of the species (g cm^−3^), D is the DBH (cm) and H is the total height (m). We used this equation (AIC = 3130, DF = 4002) because it covers a wide range of tropical trees including some species from southern Mexico.

For *Leucaena leucocephala* in FB, we developed allometric equations from the destructive sampling of 33 trees and used this equation to calculate biomass C stocks. Trees were measured in the field, cut at the ground level, and taken to the laboratory for oven drying. Dry biomass weights were then plotted to regression analysis with dasometric variables measured in the field^118^. In FB, tree heights were measured by measuring tapes or graduated sticks. Legume trees on FB are usually cut for fodder at about 1 m in height. Hence, the biomass model was developed with the base diameter (30 cm above ground level). An exponential model fitted best to our data with a determination coefficient (R^2^) of 0.95. The best fit equation is:

$${\text{AGB}}_{{\text{L}}} {\text{ = 0}}{\text{.0692 exp(1}}{\text{.0323 Ln(D}}^{{\text{2}}} {\text{H))}}$$

where AGB_L_ is the aboveground biomass (g) for *Leucaena*, D is the tree diameter 30 cm above ground level (cm), and H is the height of the tree (cm). We used this equation to calculate the *L. leucocephala* biomass in the FB of this species.

Tree root biomass was quantified with the following allometric equation^119^

$${\text{RB}} = \exp ( - 1.085 + 0.926{\text{ Ln }}({\text{AGB}})$$

where RB is the root biomass of the tree (kg dry weight), AGB is the aboveground biomass (kg dry weight).

Individual tree biomass obtained from allometric equations were summed and converted to plot-level C stocks (Mg C ha^−1^) by using the sample area (1000 m^2^) and a C fraction of 0.47. The biomass stocks from the linear measurement of trees in LF were converted to per hectare considering that 200-m fences correspond to a one-hectare plot.

We also calculated tree species richness, Shannon–Weaver diversity index (H)^120^, Sorensen similarity coefficient (CC)^121^, and Pielou evenness index (J)^122^ for each land-use by study region.

$$Shannon-Weaver index for tree diversity= - \sum_{i=1}^{s}Pi*lnPi$$

where pi is the proportion (n/N) of individuals of one particular tree species (n) divided by the total number of individuals from all species found (N), lnPi is the natural logarithm of Pi and C is the sum of the S number of species.

$$Sorensen's\;similarity\;coefficient\;(CC) = \frac{{2C}}{{S1 + S2}}$$

where C is the number of species in common between PF and the respective land-use, S1 is the total number of species found in PF, and S2 is the total number of species found in the given land-use. CC gives a value between 0 and 1, the value closer to 1 denotes that the tree species composition is more similar to PF. CC was used to measure the degree to which selected land-uses share PF (native) species.

$$Pielou's\;evenness\;index\left( J \right) = \frac{H}{{ln(S)}}$$

where H is the Shannon–Weaver index and S is the total number of tree species in the respective land-use. The result ranges from 0.0 to 1.0 with 1.0 reflecting total evenness. The evenness index (J) is low when only a few species are more abundant than others.

### Grass biomass

In OP, FB, DT, SF, and PF, we collected the grass (or understory vegetation) samples within four square frames of 1 × 1 m^2^. The 1000 m^2^ plot was first divided into four quadrants and the frames were established randomly within each quadrant. In LF, grass biomass samples were collected from four quadrants distributed to both sides of the fences within the area of canopy coverage. Quadrants (square frames) were made of PVC tubes with an inner surface area of 1 m^2^. Once the sampling quadrants were located, we cut all the grass biomass at ground level within the quadrants. The grass biomass samples were put in paper bags, labeled accordingly, weighed, and transported to the laboratory for oven drying (70 °C). The oven-dried biomass was converted to C stocks (Mg C ha^−1^) by using a C fraction of 0.46 and the sampling area (4 m^2^)^80^.

### Ground litter

The fallen leaves, small branches (≤ 1.0 cm), and their organic detritus were collected as litter mass within four 30 cm × 30 cm PVC quadrants distributed randomly within each C monitoring plot. Larger branches were sampled as deadwood material. Litter samples were collected separately in two categories depending upon the stage of decomposition. The litter samples were stored in paper bags, which were labeled with assigned personalized codes, then they were weighed, and transported to the laboratory for oven drying and chemical analysis. By using the C fraction and sample area, we extrapolated the litter C stock to Mg C per ha.

### Deadwood carbon

We used the line intersection method sampling fallen dead wood materials (> 1 cm diameter) by measuring the diameter of wood pieces across four transects of 25 m each on the borderline of each 1000 m^2^ C monitoring plot^123^. The volume of wood was calculated as:

$$V=\frac{{\pi }^{2}}{8L}{\sum }_{i=1}^{n}{d}_{i}^{2}$$

where, V is the volume of the wood (m^3^), di is the diameter (cm) of the piece of wood i, and L is the length (m) of the transect (25 m). In LF, only two transects were used. Besides, the volume of the deadwood fence (poles) was calculated by measuring the diameter and height. The deadwood mass was obtained by multiplying the volume with wood density according to the state of decomposition. A fraction of 0.47 was used to convert deadwood mass to C.

### Fine root biomass

Fine root biomass was sampled by using a 5.5 cm diameter cylinder to the respective soil depth (0–10 cm, 10–20 cm, and 20–30 cm), separated from soil, washed, and oven-dried (70 °C) before weighing^80,85^. Root fragments greater than 2 mm in diameter were discarded from fine root biomass. Dry root biomass was then converted to per hectare value by using surface area and respective soil depth.

### Soil properties

Soil samples were collected from four random locations within the 1000 m^2^ plot. To avoid the overlapping between litter and soil samples (within the organic horizon), we sampled within the same random quadrants where litter samples were collected. Soil samples were collected separately by depth (0–10 cm, 10–20 cm, and 20–30 cm) using a cylindrical metallic corer of 5.5 cm inner diameter and 10 cm height. The cylinder was struck manually until reaching the first 10 cm of soil and carefully removed and handled to obtain a complete sample. The same procedure was applied to collect consecutive soil samples from 10 to 20 and 20–30 cm depths. We collected 24 samples per plot, 12 for chemical analysis, and the parallel 12 for soil bulk density estimations.

Before chemical analysis, we separated root and rock fragments from the soil samples, dried them at room temperature, grounded, and sieved them using a 2 mm mesh. For C concentration (%), we used the potassium dichromate digestion method followed by sucrose calibrated spectrophotometry at 600 nm wavelength^124^. For soil pH and oxidation–reduction potential (Eh), we used the potentiometric method in a 1:2 soil water solution. For bulk density calculation, soil samples were oven-dried (105 °C) for 48 h, coarse fragments (roots and rocks) were separated and weighed. Soil bulk density was calculated as the ratio of dry weight to the volume of the cylinder used for sampling (g cm^−3^). Total bulk density was calculated without separating coarse fragments while soil bulk density was calculated with coarse fragment free soil mass to evaluate the effect of rock fragments. Soil bulk density is one of the important variables in the quantification of SOC stock since it is used to quantify the total soil mass per unit area to a certain depth. Using the oven-dry weight of the separated rocks, we quantified the amount of rock per unit area (Mg ha^−1^). Soil bulk density, corresponding SOC content (%), surface area, and soil depth values were used to calculate the SOC stocks (Mg ha^−1^) as follows:

$$SOC stock \left(Mg {ha}^{-1}\right)=\frac{SOCC \times BD \times Depth\times 10000}{100}$$

where, SOCC is the soil organic carbon concentration (%); BD is the soil bulk density (g cm^−3^ or Mg m^−3^) after correcting coarse fragments; Depth is the depth of soil sample (m). SOC stocks were calculated according to the soil mass equivalent after subtracting the mass of rock fragments.

### Data analysis

We first cross-checked all data from the three regions and six land-uses by creating a pivot table to visually explore the data. Any atypical value was subjected to further review and consultation with the field team. The verified data were tested for normality of distribution by the Shapiro–Wilk W test (p > 0.05). The non-normal data were transformed (log10). Log transformed data were used for one-way ANOVA to test the significant differences between sampled land-uses. We also used factorial ANOVA to test the effect of land-use and soil depth categories for SOC content and other soil properties. Back transformed weighted means and respective 95% confidence intervals are presented in the results. Furthermore, we applied a principal component analysis (PCA) based on correlations to reduce the dimensions and check the relationship between variables such as SOC concentrations, living tree biomass, soil properties, and land-use history. Variable groupings in PCA were based on correlation. We developed a correlation matrix among variables and their significance (Supplementary Table T1). To extract the principal components, we used eigenvalues greater than one. To associate variables with components, we considered those having a correlation higher than 0.5. We applied the Varimax rotation and clustered the variables in a biplot based on their loadings. The Kaiser–Meyer–Olkin measure of sampling adequacy was 0.685 and Bartlett's test of sphericity was significant (χ^2^ value = 597.5, p < 0.01) showing that the data contain enough variance to partition into components.

### Ethics approval

This research did not use any methods that require licensing committee approval.

## Supplementary Information

Supplementary Information.

## Data Availability

Data can be obtained upon request from the corresponding author. Collection of plant biomass and soil samples were carried out with permissions from local authorities. We declare that this project was carried out in collaboration with the Mexican National Commission for Natural Protected Areas (CONANP, Spanish acronym), which is the local entity that regulate such permissions. We did not use any vertebrate animal for experimental purposes in this study. The animals in the images were to represent that the lands we sampled are used for animal grazing.
