# Supplementary material for: Silvopastoral systems and remnant forests enhance carbon storage in livestock-dominated landscapes in Mexico
Source: Sci Rep. 2022 Oct 6;12:16769. doi: 10.1038/s41598-022-21089-4 (PMC9537417; doi:10.1038/s41598-022-21089-4)
Supplement: Supplementary file 1 — Supplementary Information. [file 41598_2022_21089_MOESM1_ESM.docx]

Supplementary information

**Silvopastoral systems and remnant forests enhance carbon storage in livestock-dominated landscapes in Mexico**

Deb Raj Aryal^1,2^*, Danilo Enrique Morales-Ruiz^2^, Susana López-Cruz^2^, César Noe Tondopó-Marroquín^2^, Alejandra Lara-Nucamendi^2^, José Antonio Jiménez-Trujillo^3^, Edwin Pérez-Sánchez^3^, Juan Edduardo Betanzos-Simon^3^, Francisco Casasola-Coto^3^, Alejandra Martínez-Salinas^3^, Claudia Janeth Sepúlveda-López^3^, Roselia Ramírez-Díaz^2^, Manuel Alejandro La O Arias^2^, Francisco Guevara-Hernández^2^, René Pinto-Ruiz^2^, Muhammad Ibrahim^3^

*Author affiliations*

*^1^Consejo Nacional de Ciencia y Tecnología,* C.P. 03940, *Ciudad de México, Mexico*

*^2^Universidad Autónoma de Chiapas, Facultad de Ciencias Agronómicas, Villaflores, C.P. 30470 Chiapas, Mexico*

*^3^CATIE - Centro Agronómico Tropical de Investigación y Enseñanza, 30501, Turrialba, Costa Rica*

*Corresponding author: debraj.aryal@hotmail.com


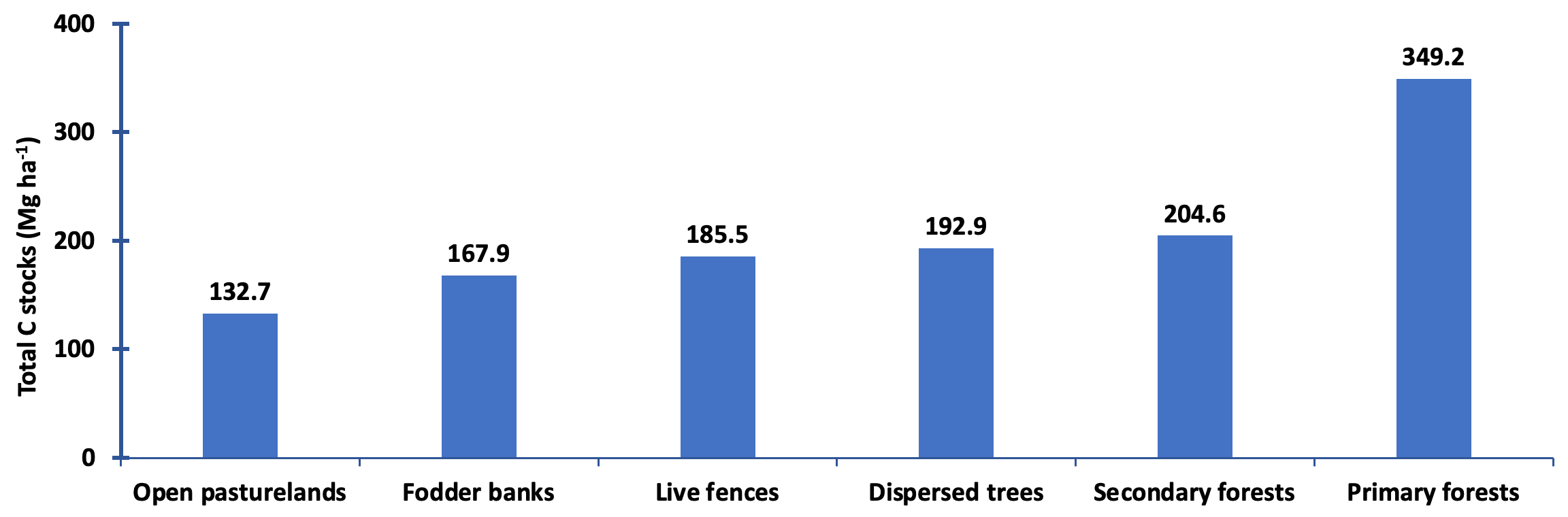


**Supplementary Figure F1**: Total carbon stocks (Mg ha^-1^) across land-use gradient. Open pasturelands are conventional grassland monocultures, while fodder banks, live fences, and dispersed trees are silvopastoral systems.

**Supplementary Table T1**: Correlation coefficients between variables. BD = bulk density (g cm^-3^), Eh = redox potential (mV), SOC% = soil organic carbon concentrations (%), LU = land-use, Rock_frag = amount of rock fragments in the soil, AGB = above-ground biomass, Landuse = the gradient of land-uses studied. *Significant at p<0.05, **significant at p<0.01, ns= non-significant.


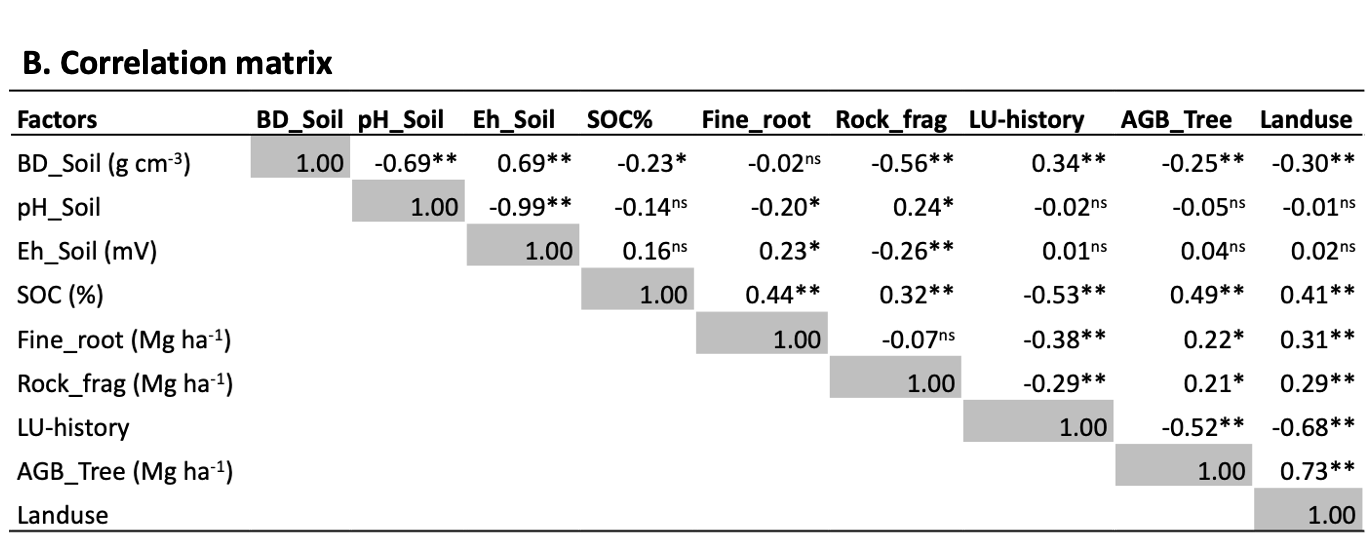


**Supplementary Table T2.** Biophysical and management characteristics of the study sites.

| **Characteristics** | **Jalisco** | **Campeche** | **Chiapas** |
| --- | --- | --- | --- |
| Geographical regions | Pacific coastal watershed, Jalisco | Southern Yucatan Peninsula, Campeche | Sierra Madre de Chiapas |
|  |  |  |  |
| Sampled municipalities | Autlán de Navarro, Tuxacuesco, El Limón, Casimiro Castillo, La Huerta, and Villa Purificación | Escarcega and Champoton | Villaflores, Jiquipilas, Cintalapa, Arriaga, and Tonala |
| Elevation range | 200 – 1300 masl. | 0 - 150 masl. | 200 – 1200 masl. |
| Climate | Tropical subhumid to semi-arid | Tropical humid to sub-humid | Tropical sub-humid to temperate humid |
| Mean annual temperature | 24°C | 25°C | 21°C |
| Average annual precipitation (mm) | 900 mm | 1600 mm | 2000 mm |
| Rainy months | June to September | May to October | May to October |
| Dominant soil types (WRB classification) | Regosols, Cambisols, and Phaeozems | Leptosols, Phaeozems, and  Vertisols | Cambisols, Leptosols, Regosols, and Luvisols |
| Soil pH | Slightly acidic to neutral (6.0 – 7.0) | Neutral to slightly alkaline (7.0 – 7.5) | Acidic (4.5 – 5.5) |
| Soil oxidation-reduction potential (Eh) | -6.6 to 50.3 | -34.4 to -0.5 | 123.3 to 147.0 |
| Dominant native vegetation | Broadleaf deciduous forests to xerophytic scrubs | Broadleaf evergreen to semi-evergreen forests | Coniferous to mixed broadleaf forests |
| Common native tree species | *Acacia farnesiana, Acacia macracantha, Albizia tomentosa, Lysiloma microphylla, Prosopis laevigata, Guazuma ulmifolia, Coursetia glandulosa, Guazuma ulmifolia* | *Piscidia piscipula, Bursera simaruba,*  *Lysiloma latisiliquum Lonchocarpus castilloi, Tabebuia rosea, Haematoxylum campechianum, Brosimum alicastrum* | *Pinus oocarpa, Quercus sps., Platymiscium dimorphandrum, Diphysa americana, Gliricidia sepium, Byrsonima crassifolia,* Enterolobium *cyclocarpum, Pithecellobium dulce* |
| Nearby natural protected areas | Reserva de la Biosfera Sierra de Manantlán, Reserva de la Biosfera Chamela-Cuixmala | Calakmul biosphere reserve, Balam-Kin and Balam-ku natural reserves | La Sepultura and Selva El Ocote biosphere reserve, Frailescana natural protection area |
| Dominant livestock farming types | Extensive bovine cattle | Extensive bovine cattle | Extensive bovine cattle |
| Average stocking rate (animal unit ha ^-1^) | 0.8 – 2.5 | 1.0 - 1.5 | 0.9 – 2.9 |
| Time after forest land-use change | 37±11 years | 25±7 years | 24±5 years |
| Sampled land-uses | Open pasturelands, fodder banks, pasturelands with dispersed trees, secondary forests | Open pasturelands, live fences, pasturelands with dispersed trees, secondary forests, primary forests | Open pasturelands, fodder banks, live fences, pasturelands with dispersed trees, secondary forests, primary forests |
| Common grass species used in silvopasture / open pastures | *Andropogon gayanus, Cynodon plectostachyus, Chloris gayana, Panicum máximum* | *Brachiaria brizantha,*  *Panicum máximum,*  *Cynodon dactylon, Brachiaria dictyoneura,*  *Cynodon plectostachyus, Sporobolus indicus* | *Brachiaria brizantha,*  *Panicum máximum,*  *Brachiaria dictyoneura,*  *Cynodon plectostachyus, Hyparrhenia rufa* |
